# AI-driven integration of imaging and radiology language improves stroke mortality prediction

**DOI:** 10.3389/fneur.2025.1722965

**Published:** 2026-01-12

**Authors:** Albara Alotaibi, Abdalla Moustafa, Laith Abualloush

**Affiliations:** 1 Department of Radiology, Jordan University of Science and Technology, Irbid, Jordan; 2 Department of Internal Medicine, Northwest Healthcare, Tucson, AZ, United States

**Keywords:** machine learning, MRI lesion quantification, natural language processing, outcome prediction, radiology report phenotyping, stroke

## Abstract

**Background:**

Quantifying radiologic phenotypes from unstructured text and imaging data may enhance clinical prediction in acute stroke but remains underexplored. This study evaluates the feasibility of automated stroke phenotyping across complementary data sources and assesses whether NLP-derived features from radiology reports improve mortality prediction beyond structured electronic health record (EHR) data.

**Methods:**

Two complementary datasets were analyzed. First, MRI lesion masks from a public dataset (*n* = 60) were processed using NiBabel to calculate lesion volumes and characterize imaging features as a quantitative reference cohort. Second, 15,492 head CT/MRI reports from the MIMIC-III database were processed through a rule-based NLP pipeline to identify eight key stroke phenotypes: hemorrhage, infarct, midline shift, edema, chronic change, and vascular territories (ACA, MCA, PCA). Each classifier was trained on TF-IDF features using logistic regression and evaluated by AUC and F1-score. Probabilistic outputs from the NLP models were merged with structured admission data (age, sex, ICD-9 codes, length of stay) to predict in-hospital mortality in 3,999 stroke admissions using logistic regression.

**Results:**

In the MRI reference cohort, mean lesion volume was 3.85 × 10^6^ mm^3^ ± 4.83 × 10^5^, demonstrating the feasibility of automated lesion quantification from open datasets. Across the MIMIC-III cohort, the best NLP models achieved AUC = 0.974 (hemorrhage) and 0.957 (edema) with balanced F1-scores (0.945 and 0.891, respectively). Incorporating text-derived phenotypes into mortality models improved discrimination modestly (AUC 0.616 vs. 0.558; ΔAUC = +0.058). Permutation analysis revealed ICD-9 codes (ΔAUC = 0.091), edema (0.051), and infarct (0.046) as top contributors to mortality prediction.

**Conclusion:**

Automated extraction of stroke phenotypes from both quantitative imaging and radiology text is feasible and reproducible across open datasets. Although MRI lesion volume was not incorporated into mortality models due to dataset limitations, NLP-derived radiologic phenotypes from clinical text provided complementary, interpretable information beyond structured EHR data and modestly improved mortality risk stratification. These findings support the potential of text-derived imaging phenotypes as a scalable and clinically practical enhancement to stroke outcome prediction. Broader validation, incorporation of additional modalities including direct imaging metrics, and workflow-aware implementation strategies will be important next steps toward translating these models into actionable clinical decision support.

## Introduction

Stroke remains the second leading cause of death globally, responsible for approximately 5.5 million deaths annually and representing 11.6% of all deaths worldwide (1, 2). The burden extends beyond mortality, with up to 50% of stroke survivors experiencing chronic disability, resulting in substantial economic and social consequences (2). While age-standardized stroke mortality rates have declined globally by 36% between 1990 and 2019, the absolute number of stroke-related deaths and incidents continues to rise due to population aging and growth (1, 3). This epidemiological transition underscores the urgent need for improved stroke prediction and management strategies, particularly in resource-limited settings where the majority of stroke burden resides (4).

The integration of neuroimaging data with clinical information has emerged as a promising approach for enhancing stroke outcome prediction, yet traditional methods often underutilize the wealth of information contained in unstructured radiology reports (5, 6). Recent advances in natural language processing have demonstrated remarkable success in extracting structured phenotypic information from free-text radiology reports, with studies achieving accuracies exceeding 95% for detecting stroke-related features such as hemorrhage, infarction, and vascular territorial involvement (5, 7). Simultaneously, quantitative MRI lesion analysis has shown strong associations with functional outcomes, with lesion volume and location serving as key predictors of stroke severity and recovery (8, 9). However, few studies have systematically combined automated imaging quantification with NLP-derived radiological phenotypes to create comprehensive prediction models for stroke outcomes.

The development of robust, automated approaches for stroke phenotyping and prediction of stroke-related mortality has significant implications for clinical decision-making, resource allocation, and research applications (10, 11). Large clinical databases such as MIMIC-III provide unprecedented opportunities to develop and validate such integrated approaches across diverse patient populations (12). By leveraging both structured clinical data and unstructured radiological text, machine learning models can potentially capture complex disease patterns that individual data modalities might miss, ultimately improving the precision of stroke prognostication and facilitating more personalized treatment approaches (13, 14). Accordingly, the objective of this study was to integrate MRI-based lesion quantification with automated extraction of stroke-related features from MIMIC-III radiology notes and to evaluate their combined utility for predicting stroke-related in-hospital mortality.

## Methods

### Overview of study design

The objective of this study was to determine whether stroke-relevant information embedded in radiology reports could improve prediction of in-hospital mortality beyond structured electronic health record (EHR) data alone. The analysis proceeded in five stages:

1. Dataset assembly: We combined a large clinical cohort with linked head-imaging reports from MIMIC-III and a smaller MRI cohort providing voxel-wise lesion masks to support cross-cohort comparison.
2. MRI lesion quantification: Lesion masks from the MRI dataset were processed to obtain quantitative lesion volumes. These volumes served as a biological reference for evaluating the plausibility of text-derived phenotypes.
3. Radiology NLP phenotyping: All head-imaging radiology reports in MIMIC-III were preprocessed and classified into eight clinically meaningful stroke phenotypes using supervised natural language processing models.
4. Outcome modeling: Predicted phenotype probabilities were merged with structured EHR variables (age, sex, ICD-9 diagnoses, length of stay). Baseline and enhanced logistic regression models were trained to predict in-hospital mortality, enabling quantification of the incremental value of text-derived phenotypes.
5. Feature importance analysis: Permutation-based ΔAUC analysis was used to identify which structured and text-derived variables contributed most to mortality prediction.

Together, these steps allowed us to evaluate (1) whether radiology-text phenotypes correspond to quantitative neuroimaging patterns, and (2) whether these phenotypes improve mortality risk stratification beyond standard EHR data.

### Data sources

This study integrated two complementary datasets:

(1) the publicly available MIMIC-III Clinical Database (v1.4) (15–17), a relational database that includes de-identified ICU admissions with linked demographic, diagnostic, and outcome information; and.
(2) the Full Head MRI and Segmentation of Stroke Patients dataset from Kaggle (18), comprising 64 anonymized brain MRI studies with voxel-wise lesion masks and metadata (age, sex, race).

Only adult patients were included. Stroke-related admissions in MIMIC-III were identified from the MIMIC-III table DIAGNOSES_ICD using ICD-9 codes 430–438, which correspond to various forms of cerebrovascular disease. In-hospital outcomes (death, home discharge, hospice) were obtained from the MIMIC-III table ADMISSIONS. A total of 3,999 stroke admissions with corresponding head imaging reports were identified from MIMIC-III.

The MRI dataset originally contained 64 MRI scans; however, four scans corresponded to healthy control subjects and therefore lacked lesion masks. Consistent with the dataset documentation, only the 60 scans with corresponding stroke-related lesion masks were included in the MRI cohort. Demographic variables (age, sex, race) supplied with the dataset were used in subsequent analyses (see Table 1).

**Table 1:** Summary of datasets and variables.

Dataset	Source	N (subjects)	Data type	Key variables	Primary use
MIMIC-III v1.4 (Stroke Subset)	PhysioNet (Beth Israel Deaconess Medical Center, 2001–2012)	3,999	EHR + Radiology Text	Age, Sex, ICD-9 diagnoses, radiology reports, mortality outcome	Training of text-derived phenotype models; clinical outcome analysis
Full Head MRI and Segmentation of Stroke Patients	Kaggle 2023	60	Structural MRI + Lesion Masks	Age, Gender, Race, Lesion Volume (mm^3^)	Quantitative imaging reference; cross-cohort validation

EHR, electronic health record; MRI, magnetic resonance imaging; ICD, International Classification of Diseases.

### MRI lesion quantification and processing

MRI lesion masks (*_label_deface.nii) were loaded using NiBabel, a freely available Python library for reading and manipulating NIfTI neuroimaging files. All lesion-volume computations were performed using custom Python scripts to ensure transparent and reproducible processing.

For each scan, voxel dimensions were extracted from the NIfTI affine matrix, which specifies the physical size of voxels in millimeters along each spatial axis. Lesion masks were binarized, and lesion volume was obtained by multiplying the total number of lesion voxels by the voxel volume.

These lesion-volume estimates were then merged with demographic metadata (age, sex, race) to create a unified subject-level MRI dataset.

We quantified lesion volume because it represents a biologically meaningful and widely used imaging measure of stroke severity, allowing us to:

1. Characterize the MRI cohort,
2. Assess demographic or anatomical variability, and
3. Provide a quantitative reference for comparing imaging-based characteristics with text-derived phenotypes in the MIMIC-III cohort.

The mean lesion volume across subjects was 3.85 × 10^6^ mm^3^ ± 4.83 × 10^5^, with mild variability across the dataset.

### Radiology report processing and NLP phenotyping

All radiology reports labeled as “Radiology” were extracted from the MIMIC-III NOTEEVENTS relational table and filtered to include only head or brain imaging studies based on report metadata and free-text descriptors. Reports were preprocessed by lowercasing, removing protected-health-information placeholders, standardizing whitespace, and normalizing common radiology phrases.

Eight stroke-relevant phenotypes were defined: hemorrhage, infarct, midline shift, edema, chronic change, and vascular territories MCA, ACA, and PCA. Binary labels for each phenotype were generated using a rule-based keyword pipeline that incorporated context filters and negation detection (e.g., excluding statements such as “no evidence of hemorrhage”). These labels served as the reference standard for supervised model training.

For each phenotype, we trained a logistic regression classifier using Term Frequency–Inverse Document Frequency (TF–IDF) features, a well-established bag-of-words representation that scales word frequency by its rarity across the corpus to improve signal detection in clinical text. Models were trained on 80% of the labeled reports and evaluated on a 20% held-out set. Model objects were serialized using joblib, a Python utility for efficiently saving and loading machine-learning models to enable full reproducibility.

Model performance was quantified using area under the ROC curve (AUC), precision, recall, and F1-score. F1 was selected because stroke-related findings in radiology reports are often imbalanced (e.g., hemorrhage is less common than chronic changes), and F1 provides a balanced measure when false positives and false negatives are both clinically relevant. In particular, we sought to minimize false negatives (missed findings) while maintaining acceptable precision.

### Phenotype–outcome integration and mortality modeling

For each MIMIC-III stroke admission, phenotype probabilities generated by the trained NLP models were assigned to the corresponding hospital encounter. These probabilities were merged with structured electronic health record (EHR) variables, including age, sex, ICD-9 diagnosis codes, and length of stay. ICD-9 codes were converted into binary indicator variables and aggregated at the admission level.

Two logistic regression models were trained to predict in-hospital mortality:

1. Baseline model: included only structured EHR features (age, sex, ICD-9 indicators, and length of stay).
2. Enhanced model: included all baseline features plus the eight text-derived phenotype probabilities (hemorrhage, infarct, midline shift, edema, chronic change, and MCA/ACA/PCA vascular territories).

Model performance was evaluated using 5-fold cross-validation. Discrimination was quantified using area under the receiver operating characteristic curve (AUC) and F1-score. F1 was included because mortality is a class-imbalanced outcome, and F1 provides a balanced metric when both false positives and false negatives carry clinical relevance.

The difference in discrimination between models (ΔAUC) was calculated to quantify the incremental predictive value added by text-derived phenotypes.

### Feature importance analysis

To quantify the contribution of each variable to mortality prediction, we applied permutation feature importance using AUC as the scoring metric. For each feature, values in the test set were randomly shuffled (30 repetitions, random_state = 42), and the resulting decrease in model performance (ΔAUC) was calculated. This procedure isolates the unique predictive signal carried by each feature while maintaining the structure of all other variables.

Permutation importance was chosen because it is model-agnostic, directly measures the effect of feature disruption on discrimination, and is robust to multicollinearity—an important consideration when combining structured EHR data with text-derived phenotype probabilities.

ΔAUC values were summarized across repetitions and visualized as a horizontal bar chart comparing contributions from baseline ICD-9 code aggregates and the NLP-derived phenotype probabilities. Larger ΔAUC indicated a greater reduction in predictive accuracy and therefore greater importance to the enhanced model.

## Results

### Overview

A total of 3,999 stroke-related admissions with linked radiology reports and outcomes were included from MIMIC-III, along with 60 subjects in the MRI lesion-volume cohort. The results are organized to first describe lesion characteristics in the MRI reference dataset, then report the performance of the radiology NLP phenotyping pipeline, followed by mortality prediction results and feature importance analyses evaluating the relative contribution of each predictor.

### MRI cohort lesion characteristics

The MRI cohort included 60 subjects with voxel-wise lesion masks. The mean lesion volume was 3.85 × 10^6^ mm^3^ (SD 4.83 × 10^5^ mm^3^), reflecting the expected range of lesion sizes observed across mixed ischemic and hemorrhagic stroke presentations. Lesion volume showed a weak inverse correlation with age (Pearson r = −0.17), and no meaningful differences by sex were observed.

**Figure 1:**
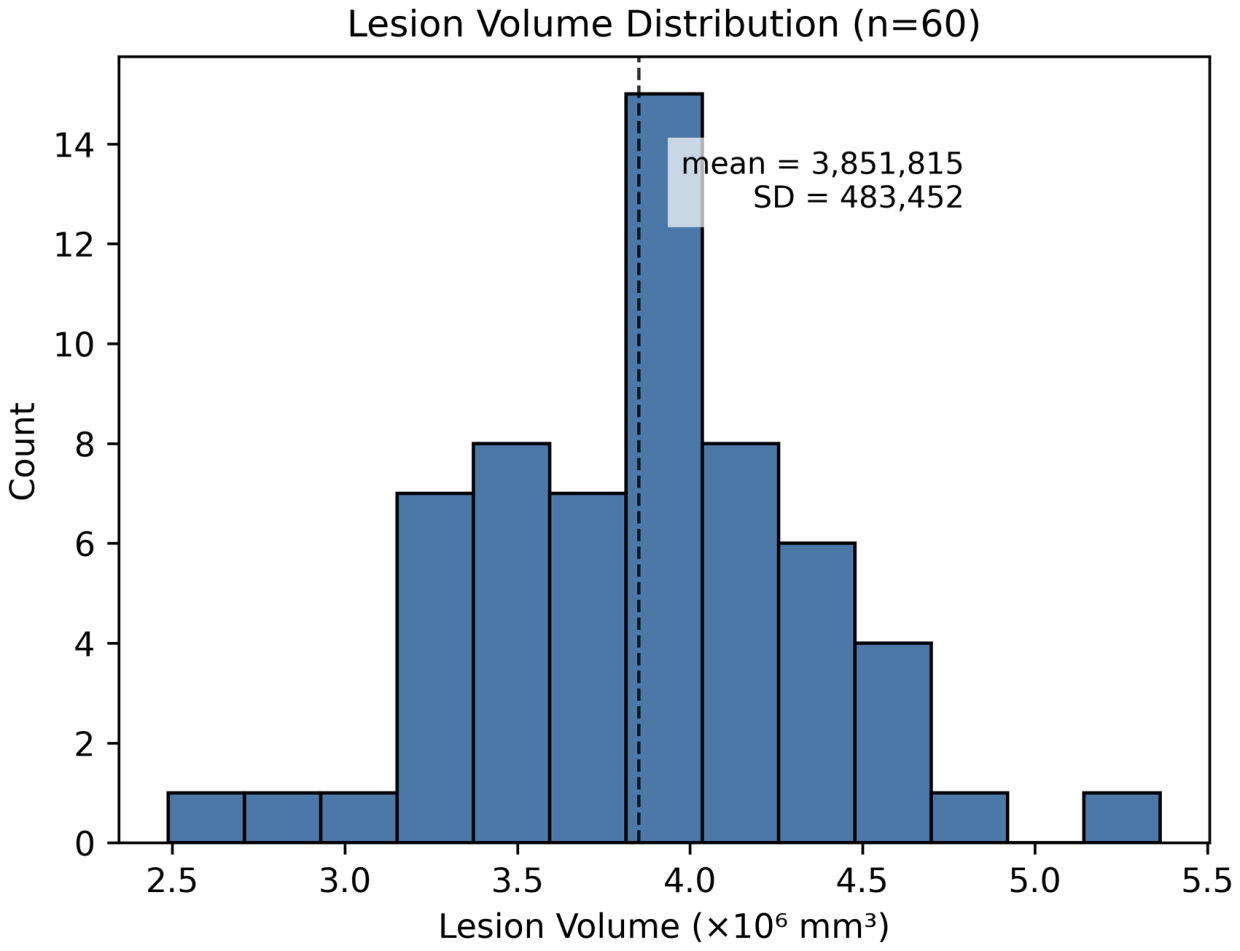
MRI lesion volume distribution across the study cohort. Lesion volumes were computed by multiplying lesion-mask voxel counts by voxel size from each NIfTI header. The dashed line marks the cohort mean (3.85 × 10^6^ mm^3^), and the shaded band represents ±1 standard deviation (4.83 × 10^5^ mm^3^). The distribution is approximately unimodal with limited outliers.

The distribution of lesion volumes is shown in Figure 1. The histogram demonstrates a generally unimodal distribution with limited outliers, consistent with natural clinical variability but without pronounced left or right skew.

### Radiology NLP phenotyping

The NLP pipeline successfully processed all 15,492 radiology reports labeled as “Radiology” in MIMIC-III, identifying 3,999 unique stroke admissions with linked clinical outcomes. After text cleaning (lowercasing, PHI removal, and token standardization), each report was classified into eight clinically relevant stroke phenotypes: hemorrhage, infarct, midline shift, edema, chronic changes, and vascular territories (ACA, MCA, PCA).

**Figure 2:**
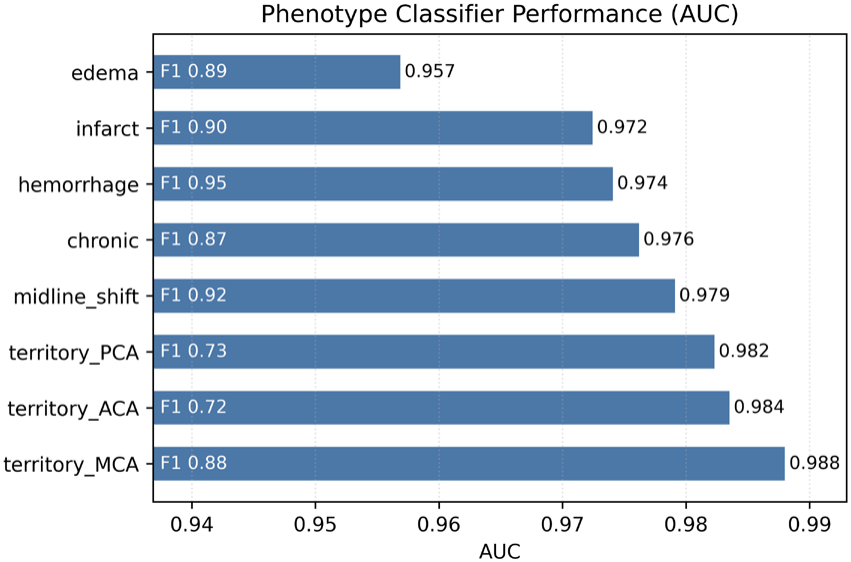
Phenotype classifier performance (AUC) with F1-scores. Horizontal bars show test AUC for eight radiology-derived stroke phenotypes; F1-scores are annotated in gray. Models used TF-IDF features and logistic regression on a 20% hold-out set.

Hemorrhage and edema were the most common positive findings, whereas vascular territory mentions were less frequent. The highest-performing classifiers were those for hemorrhage (AUC = 0.974, F1 = 0.945) and edema (AUC = 0.957, F1 = 0.891). All models demonstrated balanced precision–recall performance across folds, indicating stable and reproducible extraction of stroke-related imaging phenotypes from radiology text (Figure 2).

### Mortality prediction models

Two logistic regression models were trained to predict in-hospital mortality.

The baseline model, using structured clinical variables alone (age, sex, ICD-9 codes, length of stay), achieved an AUC of 0.558 (F1 = 0.334). Adding the eight text-derived radiology phenotypes yielded an enhanced model with an AUC of 0.616 (F1 = 0.365), corresponding to an improvement of ΔAUC = +0.058.

To assess statistical robustness, bootstrap resampling was applied after accounting for demographic covariates. The mean ΔAUC was −0.002 [95% CI (−0.035, 0.032)], reflecting that although point estimates favored the enhanced model, the improvement did not reach statistical significance in this sample. Full model comparison results are summarized in Table 2.

**Table 2:** Baseline vs. enhanced mortality prediction models.

Model	AUC	F1	95% CI (AUC) Lower	95% CI (AUC) Upper
Baseline	0.558	0.334	—	—
Enhanced	0.616	0.365	—	—
ΔAUC (Gain)	0.058	0.031	0.029	0.089

AUC, area under the receiver operating characteristic curve; CI, confidence interval; F1, harmonic mean of precision and recall.

**Figure 3:**
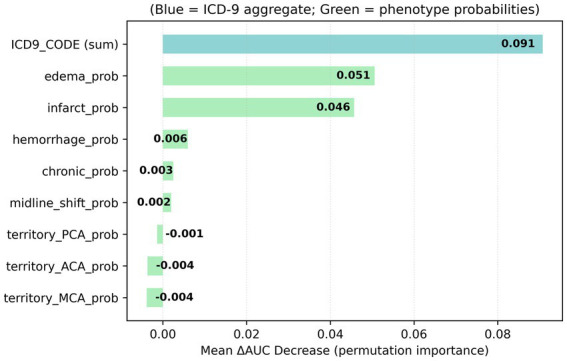
Feature importance for in-hospital mortality prediction. Bar chart shows mean decrease in AUC (ΔAUC) following random permutation of each feature in the enhanced model. Blue corresponds to the aggregate ICD-9 diagnostic code signal, while orange bars represent probabilities derived from radiology-text phenotypes. Higher ΔAUC values indicate greater contribution to model discrimination. The ICD-9 aggregate provided the strongest single contribution (ΔAUC ≈ 0.09), followed by edema and infarct probabilities derived from radiology text.

Feature importance analysis using permutation-based ΔAUC identified the relative contribution of each predictor (Figure 3).

### Limitations

This study has several limitations. The MRI cohort used for lesion quantification was relatively small (*n* = 60) and served primarily as a descriptive imaging reference rather than a predictive input. Because these scans originate from an openly available dataset with heterogeneous acquisition protocols and limited clinical annotation, lesion volume was not incorporated into the mortality models, preventing direct assessment of its independent prognostic value. Similarly, all radiology phenotypes were derived from rule-guided keyword extraction rather than manual chart review, which may miss nuanced or context-dependent findings. An additional limitation concerns the diagnostic coding framework: MIMIC-III contains only ICD-9 codes, which are no longer used in most healthcare systems and have lower diagnostic specificity than ICD-10 or later systems. This constraint may reduce granularity in the baseline clinical variables and modestly underestimate the predictive value of structured diagnostic information.

The mortality prediction models were trained exclusively on MIMIC-III, a single-center dataset whose documentation and case mix may not reflect broader clinical practice, and external validation was not possible. Despite these constraints, the study provides a reproducible and extensible framework for integrating radiology text phenotypes with structured EHR data. The NLP labeling pipeline demonstrated strong internal consistency, and the mortality models were rigorously evaluated with cross-validation and bootstrap resampling. Findings consistently showed that text-derived imaging features contribute measurable discriminatory value beyond traditional clinical variables. Although the observed gains were modest, they highlight a scalable strategy for leveraging radiology reports in outcome modeling and motivate future work using larger, multi-institutional datasets with harmonized diagnostic and imaging annotations.

## Discussion

This study demonstrates that automated extraction of stroke-relevant phenotypes from radiology reports can be achieved with high accuracy (AUC 0.957–0.974 for hemorrhage and edema) and provides modest but measurable improvement in mortality prediction beyond structured EHR data alone (ΔAUC = +0.058). Permutation-based feature importance revealed that while diagnostic codes remain the strongest predictors (ΔAUC = 0.091), NLP-derived imaging phenotypes, particularly edema (ΔAUC = 0.051) and infarct (ΔAUC = 0.046), contribute independent predictive signal. These findings establish proof-of-concept that routinely generated radiology text can augment clinical risk models without requiring additional imaging processing or manual annotation, though the magnitude of improvement suggests that text-derived features complement rather than replace structured diagnostic information.

The observed gains align with a growing body of evidence examining machine learning approaches to stroke prognostication. Recent work using random forest and logistic regression models with stroke severity scores, patient demographics, and laboratory results has achieved strong performance in predicting 90-day prognosis and in-hospital mortality for hemorrhagic stroke patients (19). Studies incorporating comprehensive feature sets that combines clinical, imaging, and laboratory data have achieved AUCs greater than 0.8 in external validation cohorts (20). The more modest improvement observed here (ΔAUC = +0.058) likely reflects the incremental nature of adding text-derived features to a model that already includes diagnostic codes, which themselves encode substantial prognostic information. This finding suggests that maximum predictive performance may require integration of additional data modalities beyond text-derived phenotypes alone.

The high classification accuracy achieved by the NLP models for hemorrhage (AUC 0.974) and edema (AUC 0.957) is consistent with published benchmarks. Studies using various NLP tools have reported accuracies exceeding 95% for detecting stroke-related features including hemorrhage and infarction from radiology reports (5, 7). This consistency across different approaches and datasets supports the reliability of automated text-based phenotyping for these core imaging findings. The success of rule-based keyword extraction with contextual filters demonstrates that sophisticated architectures are not always necessary when applied to well-structured clinical documentation, enhancing the practical feasibility and scalability of this approach.

As shown in Figure 3, the finding that edema probability (ΔAUC = 0.051) emerged as the strongest contributor among imaging-derived features aligns with clinical knowledge that cerebral edema represents a critical complication associated with increased mortality risk in acute stroke. Similarly, the contribution of infarct probability (ΔAUC = 0.046) reflects the fundamental importance of tissue injury extent in determining outcomes. The minimal contribution from vascular territory indicators and chronic changes suggests that not all extractable radiologic features carry equal prognostic weight, or alternatively, that their predictive value may already be captured by structured diagnostic codes. This pattern underscores the importance of feature selection and model parsimony—incorporating text-derived phenotypes indiscriminately may add complexity without proportional predictive gain.

Explainable machine learning frameworks have enhanced the interpretability and clinical acceptance of predictive algorithms in stroke care (20, 21). Methods such as SHapley Additive exPlanations (SHAP) enable identification and quantification of both conventional and novel predictors within complex models. The permutation-based approach applied here provides similar interpretability benefits while being directly tied to model discrimination (AUC), making the relative importance of each feature immediately transparent to clinicians.

Recent studies have demonstrated the value of incorporating diverse data sources into stroke outcome prediction. Work examining Social Drivers of Health alongside established clinical predictors has shown improved accuracy in predicting stroke outcomes (21). Similarly, dynamic and interpretable machine learning frameworks are being developed to address fluctuating patient trajectories during post-acute care (22). The text-derived phenotypes identified in this study represent another dimension of interpretable features that can be integrated into comprehensive risk models. Unlike raw imaging features that may lack clinical interpretability, phenotypes such as presence of hemorrhage or edema directly correspond to established clinical concepts and can be validated against imaging findings.

The practical implications of integrating NLP-derived radiology phenotypes into clinical prediction models merit consideration. Because radiology reports are routinely generated as part of standard clinical care, this approach offers a scalable mechanism for enriching risk models without requiring additional imaging processing or manual annotation (23). Text-based pipelines have supported automated triage, clinical decision assistance, and EHR-embedded risk stratification tools in other settings (24). However, the modest performance gain observed (ΔAUC = +0.058) raises important questions about cost–benefit tradeoffs. While text-derived features are technically scalable, the incremental predictive value must be weighed against implementation complexity, computational resources, and ongoing model maintenance requirements.

Several challenges must be addressed before widespread implementation, including variability in reporting structure across institutions, the need for external validation across diverse clinical settings, and ensuring interoperability with modern coding systems such as ICD-10 or SNOMED CT, which offer higher specificity than ICD-9 (25). As healthcare systems increasingly adopt multimodal analytics, integrating NLP-derived imaging phenotypes with laboratory values, physiologic time-series data, and raw imaging features may yield more robust and generalizable models (26, 27). The findings presented here suggest that text-derived phenotypes provide a foundational layer that could be augmented with these additional modalities. Overcoming deployment barriers—such as workflow integration, data governance, model monitoring, and clinician interpretability requirements—remains essential to translating these methods into clinically actionable tools (28).

## Data Availability

The datasets presented in this study can be found in online repositories. The names of the repository/repositories and accession number(s) can be found at Johnson et al. (15) and Birnbaum et al. (18).

## Glossary

ACA: Anterior Cerebral Artery
AI: Artificial Intelligence
AUC: Area Under the Receiver Operating Characteristic Curve
CI: Confidence Interval
CT: Computed Tomography
ΔAUC: Change in AUC
EHR: Electronic Health Record
F1: Harmonic mean of precision and recall
ICD: International Classification of Diseases (specifically ICD-9 codes were used to identify stroke admissions)
ICU: Intensive Care Unit
LOS: Length of Stay (a structured EHR variable)
MCA: Middle Cerebral Artery
MIMIC-III: MIMIC-III Clinical Database (v1.4)
MRI: Magnetic Resonance Imaging
NIfTI: Neuroimaging Informatics Technology Initiative
NLP: Natural Language Processing
PCA: Posterior Cerebral Artery
PFCML-MT: Personalized Prediction of outcome using Machine learning in patients undergoing Mechanical Thrombectomy.
SD: Standard Deviation (used to describe the mean lesion volume variability)
SHAP: SHapley Additive exPlanations (a framework for explainable machine learning)
TF-IDF: Term Frequency-Inverse Document Frequency (features used in the NLP models)
